# Protocol for an undergraduate student-led scoping review of methods used to conduct inclusive focus groups with autistic adolescents

**DOI:** 10.1371/journal.pone.0338882

**Published:** 2025-12-16

**Authors:** Nivedita Medum, Trevor Stocker, Mary Finkler, Júlia Hellín López, Noah Edwards, Makenna Sommers, Morgan Gaston, Ella Marie Johnson, Amanda Schadler, Josh Byars, Julia Griese, Celeste Campos-Castillo

**Affiliations:** 1 Lyman Briggs College, Michigan State University, East Lansing, Michigan, United States of America; 2 Department of Physiology, Michigan State University, East Lansing, Michigan, United States of America; 3 Department of Media and Information, Michigan State University, East Lansing, Michigan, United States of America; 4 College of Natural Science, Michigan State University, East Lansing, Michigan, United States of America; 5 Department of Neuroscience and Psychology, Michigan State University, East Lansing, Michigan, United States of America; 6 Department of Psychology, Michigan State University, East Lansing, Michigan, United States of America; 7 Department of Statistics and Probability, Michigan State University, East Lansing, Michigan, United States of America; Nagoya University: Nagoya Daigaku, JAPAN

## Abstract

Although focus groups gather early-stage input effectively, our initial literature review found few focus group studies conducted with autistic adolescents (ages 12–19), despite the potential for focus groups to provide a safe, peer-based setting that encourages autistic adolescent engagement in research. Scoping reviews of focus groups for children and people with disabilities exist, but not for autistic adolescents. We aim to fill this gap. Consequently, we plan to conduct a scoping review to identify the methods used to design inclusive focus groups for autistic adolescents. Because few relevant studies exist, we describe steps to search both academic databases and online sources (X, YouTube, Google). We detail how we will leverage our team composition, which is led by a large group of undergraduate students, some of whom are neurodiverse, to enhance the rigor and reproducibility of the scoping review. These steps include accounting for algorithms personalizing search results from online sources and the risk of encountering false information that could cause harm. We will analyze the results to show 1) the extent to which focus groups on autistic adolescents are conducted with autistic adolescents; 2) characteristics of autistic adolescents included in focus groups and underrepresented populations; 3) steps taken to design accessible focus groups for autistic adolescents; 4) which methods were feasible for and acceptable to autistic adolescents. The results of our scoping review will be an important step toward including input from autistic adolescents in the early stages of a project and, more broadly, in the research process.

## Introduction

Focus groups (FGs), which are typically used at the first stage of a project to gather input that can enhance the project’s effectiveness [1–3], involve people gathering to discuss a specific issue and providing their perspectives and experiences. Scholars and practitioners from diverse fields have found FGs beneficial for encouraging, sharing, and building on each FG participant’s ideas, thereby soliciting input that would be unobtainable through other methods [4–7]. Moreover, while the group setting may at times inhibit participants from responding to sensitive questions, it can also be beneficial because they may feel less pressure to respond to every question. Specifically, research suggests this benefit makes FGs ideal for children, because the presence of other children creates a safe peer environment that addresses the power imbalance that occurs when a single child is interviewed by a single adult interviewer [8,9].

Among the subsets of children for which FGs may be particularly beneficial for gathering input are autistic adolescents (12–19 year-olds). Studies observe that social communication among autistic children declines as they enter adolescence and increases for some as they enter adulthood [10–12]. Adolescence is a developmental period in which the social environment becomes increasingly complex (e.g., the increased importance of peers and obtaining their positive evaluation), and while this can disadvantage autistic adolescents when socializing with others [11], it also presents an opportunity to enhance their development [12].

To identify how and when FGs may be beneficial, we adopt a neurodiversity affirming framework [12–14], which reframes autistic individuals as “neurodiverse” by shifting the focus from deficits that need to be corrected toward recognizing variants in abilities as strengths. The framework maintains that coordinating an autistic person’s fluency in an environment is accomplished by *accommodating* them [15]. For example, other research on group interactions among autistic adolescents proposes that factors such as modality (online versus in-person), incorporation of special interests (the deeply meaningful interests of an autistic individual), and the presence of caregivers or peer mentors shape their ability to engage [12,16–20], suggesting the need to consider these when designing accessible FGs. Consequently, designing inclusive FGs entails designing settings that enable diverse groups of autistic adolescents to enroll and share their perspectives.

Unfortunately, from an initial review of the peer-reviewed scientific literature to identify best practices for creating accessible FGs for autistic adolescents, we noted few studies conducting FGs *with* autistic adolescents but several *on* autistic adolescents that were with other stakeholder groups (e.g., caregivers, teachers). This is consistent with the findings of a previous review on a related topic, which showed children with disabilities are rarely included in research about children with disabilities [21]. Moreover, in our initial review we noted that among studies conducting FGs with autistic adolescents, few detailed steps that researchers undertook to design accessible settings. Without these details, scholars seeking to conduct FGs with autistic adolescents in the future have few models to follow, thereby hampering efforts to enhance the inclusion of autistic adolescents in research.

The aim of our research is to provide evidence-based guidelines for designing inclusive focus groups for adolescents, which we operationalize as designs that increase participation rates for this group and empower them to share their perspectives. We will conduct a scoping review of methods used to design inclusive FGs. Because we anticipate a low number of research studies in academic databases that fit the criteria for inclusion (described momentarily), we will also conduct an online search of Google Search, X (formerly known as Twitter), and YouTube to identify best practices that may be shared by practitioners working with autistic adolescents who may not publish in academic outlets. We anticipate the online search would yield (either directly or via links to additional sources) blogs, personal and organizational webpages, and additional research studies not captured in academic databases (e.g., works in progress). We considered both TikTok and Reddit as part of our unconventional sources, but decided against them because they did not meet our research needs. A search of TikTok yielded primarily content that was unrelated to focus groups or autistic youth. Moreover, due in part to its short-form videos, the content lacked methodological detail or source verification. Similarly, Reddit lacked a way to check the credibility of posts, specifically because users post content pseudonymously (with usernames they create).

Adding an online search introduces concerns, such as the lack of reproducibility of the search due to algorithms that personalize search results [22–24]. Moreover, incorporating ideas into the review that have not undergone peer-review risks spreading potentially harmful beliefs and practices. Social media is fraught with false information, and this includes topics about autism [25–27]. Accordingly, we will document our plan for searching and evaluating the credibility of the results obtained from these three sources. Our team is comprised of 11 undergraduate students enrolled in a first-year Honors seminar, and thus we will take steps to leverage the size of our team and enhance their expertise [28]. A focus of the Honors seminar was to design accessible research tools for autistic adolescents. An additional benefit of our team is that among the Honors students are neurodiverse individuals.

## Methods

To better understand how focus groups are designed to be inclusive for autistic adolescents, we plan to complete a scoping review to examine the literature. Before detailing our planned steps, we describe how the undergraduate Honors students prepared to lead the scoping review and develop the protocol.

### Student preparation

Prior to deciding on a scoping review, the students received a hands-on lesson that provided an overview of the benefits and drawbacks of different research methods, with an eye toward conveying foundational concepts of research design. The lesson was taught by a professor with a background in sociology and ten years of experience teaching research methods to undergraduate students.

Surveys were introduced by reviewing the findings of election polls. The concepts of validity and reliability were defined and applied to understand how self-report methods impact the ability to predict election outcomes. To demonstrate how question design shapes validity and reliability, students were asked to complete a scale individually and then joined a classroom discussion critiquing question wording and how this shapes validity and reliability. This portion of the lesson was included to equip students with a background on how to produce valid and reliable research results. Experiments were introduced and the classroom conducted probability exercises to understand how random selection and random assignment enable generalization and inferring causality. This portion of the lesson was included to explain the importance of minimizing systematic bias (i.e., non-randomness) to strengthen inferences. Lastly, the lesson introduced interviews and compared the benefits and drawbacks of individual versus focus group interviews. The classroom held a discussion to identify instances in which one design may be more appropriate over the other for encouraging participation rates and sharing personal experiences. This portion of the lesson was included to provide a background on the focal design for the review, which was focus groups. The discussion then turned to brainstorming how to design accessible focus groups for autistic adolescents.

A second lesson introduced three different types of reviews: narrative review, scoping review, and systematic review. The students were assigned to read three articles that discussed the differences between the three types and the potential role for undergraduate students in the process [28–30]. The students were also given two discussion questions in advance to guide their reading: 1) What are the differences between systematic reviews, scoping reviews, and narrative reviews? 2) What are some of the benefits for undergraduate students who assist in conducting reviews? What are some of the downsides?

During discussion, students considered the different types and began developing a working plan for how to structure their work. Based on a reading [29], they requested additional assistance from a more senior student with research experience to facilitate answering their questions while they undertake the review. Subsequently, a master’s student was brought in to assist. The students concluded it was ideal to divide themselves into teams. The students believed that the structure of having teams, the professor, and a more senior student would encourage peer-to-peer learning and increase access to expert assistance.

At the next class meeting, the 11 students were divided into three teams, with one team (n = 3) tasked with learning and teaching the rest of the students how to design and write a protocol for a scoping review, a second (n = 4) tasked with developing the plan for the online search, and a third (n = 4) tasked with developing the plan for searching academic databases. The protocol was refined over the next 6 class meetings, with teams presenting their work-in-progress to the entire classroom for feedback at the end of each meeting. The remainder of the Methods section details the final plan.

### Overview

We chose a scoping review because it is appropriate when the objective is to identify how research is conducted with a specific population, [31,32]. The protocol was developed to accord with the Preferred Reporting Items for Systematic Reviews and Meta-analysis for Protocols (PRISMA-P) and the PRISMA extension for scoping reviews (PRISMA-ScR) guidelines [33]. We will follow the modified Arksey and O’Malley scoping review frameworks and the Joanna Briggs Institute Manual for scoping review studies framework [33–35]. The proposed framework includes: 1) developing a research question; 2) developing a search strategy, 3) evidence screening and selection; 4) data extraction; 5) data analysis; and 6) consultation. The preliminary literature search began October 2024 and we expect the review to be completed Spring 2026.

### Stage 1: Developing a research question

The full class developed and clarified the research questions. This scoping review aims to understand methods to design focus groups to make them inclusive for autistic adolescents. This includes mapping inclusion, specifically the extent to which autistic adolescents are included and the background characteristics of those who are, along with the accommodations described and how autistic adolescents responded to them. By documenting this information, we aim to inform others about best practices and spur greater research in partnership with autistic adolescents. The following research questions will be addressed:

1. To what extent do focus groups that were conducted to understand autistic adolescents include autistic adolescents?
2. Of the autistic adolescents who were included in focus groups, what background characteristics are underrepresented?
3. Among focus groups that included autistic adolescents, what accommodations were used to make them accessible?
4. What accommodations were reported as feasible for and acceptable to autistic adolescents?

### Stage 2: Developing a search strategy

Our search strategy was developed and refined based on an initial search in October 2024. For example, we considered including “neurodiverse” in the keywords used, but discovered it added populations irrelevant to our research questions. We also identified a need to use a search strategy that differed slightly for one of the databases.

We will search for peer-reviewed journal articles and conference proceedings in the following databases: ACM Digital Library, MEDLINE, PsycINFO, PubMed. The following keywords, wildcards, and Boolean operators will be used to capture the research design (focus group) and population (autistic adolescents) of interest: (“focus group*”) AND (“autism” OR “autistic” OR “ASD” OR “Asperger*” OR “pervasive developmental disorder”) AND (“adolescent*” OR “child*” OR “young adult*” OR “middle school” OR “high school” OR “pediatric” OR “youth” OR “secondary school”). For MEDLINE, PsycINFO, and PubMed, we restricted the search to the titles and abstract, while for ACM Digital Library we searched the full text to accommodate that some conference proceedings had short or unstructured abstracts that did not detail the study’s research method or population.

The online search will use similar keywords. We will search Google Search, X, and YouTube. Because algorithms can optimize information retrieval by matching users’ information needs with the available content [36], we devised a plan to leverage the architecture of each online source’s algorithm. Consequently, we familiarized ourselves with prior scholarship identifying concerns and best practices for searching these sources [22–24,37,38], and the latest descriptions of the algorithms to develop the search strategy. A summary of their algorithms and available search filters (as of January 2025) is shown in Table 1, along with the sources we used to compile this information.

**Table 1:** Summary of algorithms and available search filters for online sources as of January 2025.

Online source	Summary of algorithm	Sorting mechanisms	Source for information
Google Search	Reviews the meaning of the words entered into the query to identify related words. Results shown are ranked based on the relevance of content presented to the meaning of the words, the usefulness and credibility of the content, accessibility of the content, and the user’s account settings. An account setting that may affect search results includes the user’s geographic location and past search history.	Time filters that limit results to content that has been posted within a certain time frame and Safe Search which censors inappropriate content.	[39]
YouTube	Videos are considered relevant based on videos the user has enjoyed in the past, videos that have been watched together by other users, and the amount of content relevant to a particular channel or topic that has been viewed. YouTube also recommends videos based on their performance such as whether users choose to watch, ignore, or click “not interested” on a video, the watch time of users, the like-to-dislike ratios for videos, and post-watch survey results that ask users whether the video was a good recommendation. Upload date is based on the date in which the video was uploaded. View count is the number of times the video has been viewed. Rating is the aggregate rating of the video by viewers.	Relevancy, upload date, view count, rating	[40]
X	Top posts are determined by engagement, health, and relevance scores. Engagement scores are influenced by how much an users positively interact with a post and its author. If a post and the author‘s history of posts are popular and trending (e.g., their likes, reposts, and replies are high), their engagement score will increase. Health scores are influenced by how much users negatively interact with a post and its author. If a post and its author are not well received and liked among other users, its health score will be low. A relevance score is based on how pertinent the content is to the search query and the timing in which the content was posted. Latest posts are determined based on the timing in which the content was posted. Posts by relevant users are determined by users’ popularity and engagement score. Posts with media are posts that include media. Lists are recommended based on the content of the lists.	Top posts, latest posts, posts by relevant users, posts with media, lists with content relevant to search	[41]

As can be seen in Table 1, the algorithms either present results (Google Search) or provide sorting mechanisms (YouTube, X) that optimize identifying relevant sources. Table 1 shows that among the ways the algorithms rank results is by considering how other users have evaluated the content, which is a classic feature of information search algorithms to facilitate identifying content that is likely credible [42,43]. The logic behind such ranking is that the crowd of users collectively assess the veracity of the information conveyed within the online content, similar to how Wikipedia and Community Notes leverage the crowd of users to curate content posted so that information is modestly accurate [44,45]. We describe in Step 3 and Step 4 the additional steps we take to screen for credible content. Despite the algorithms providing benefits for our online search, the descriptions of each in Table 1 confirm concerns that all three sources personalize results, which threatens the rigor and reproducibility of scoping reviews [22–24]. Accordingly, we leveraged the size of our research team to develop a search strategy that leverages the algorithms to optimize our search while reducing the influence of personalization.

Each source will be searched by a team of three researchers, who will compare their search results to each other to identify any differences in the results and determine potential reasons for differences. For example, if two team members obtain the exact same results, but the third does not, this is likely due to the third team member not following steps. If it is determined that steps were followed by all three team members, then the discrepancy in results is likely to due to unknown features of the algorithm and discrepant results will be included.

Each member of the team of three researchers conducting the online search will follow these steps:

1. Log out of personal accounts for the source being used or erase account history. If this is X, create a new account and do not like or follow any content.
2. Erase cookies and search history from browser being used.
3. Enter search terms exactly as listed, paying attention to capitalization and spacing between terms. If this is X, convert the search terms into hashtags to identify additional results.
4. For YouTube and X, sort results by relevancy (‘Relevancy’ for YouTube, ‘Top posts’ for X).
5. Compare the first five results to those of the other two team members.
6. Repeat with the next combination of search terms.

As noted in the steps, we will stop after the first 5 results for each combination of search terms. During our initial search, we tested other recommended stopping rules [37], such as selecting the first 10 results. However, as with the search within peer-reviewed databases, we noted few relevant results. We also noted saturation (same content appearing in the search results for each combination of search terms) when testing different search terms after the first five results. Accordingly, scholars seeking to apply our procedures for other reviews may need to consider other stopping rules that are more suitable to their search results.

### Stage 3: Evidence screening and selection

We will use Endnote to remove duplicate peer-reviewed articles and Excel to complete the screening of peer-reviewed articles and results from the online sources. For the articles, two team members who are undergraduate students will independently screen and review 10 articles, first completing title/abstract screening (level 1-screening) and then full-text article screening (level 2-screening). Any discrepancies in screening will be discussed by the team members and, if necessary, resolved by consulting with a third team member. The process of comparing screening and reviewing sets of 10 articles will repeat until at least 75% agreement is reached. At that point, the undergraduate student team members will split the remaining articles and screen and review them independently.

Results from searching for peer-reviewed articles and the online search will be included and excluded based on criteria listed in Table 2. Using Excel, we will also track the number of studies conducting focus groups about autistic adolescents that included other stakeholder groups (e.g., caregivers, teachers) to document the extent to which focus groups about autistic adolescents include autistic adolescents.

**Table 2:** Inclusion and exclusion criteria.

Criteria	Inclusion	Exclusion
Population	Participation in the focus group by at least one autistic adolescent between the ages of 12 and 19.	An autistic adolescent between the ages of 12 and 19 was not included in the focus group.
Concept	A topic of discussion in the focus group is about autistic adolescents between the ages of 12 and 19.	The focus group did not discuss a topic related to autistic adolescents between the ages of 12 and 19.
Design	Focus group with at least two members.	Not a focus group with at least two members.
Time period	Any time period.	
Setting	Any setting will be considered.	
Language	Sources available in English.	Sources not available in English.

Additionally, for the online search, we will screen for the credibility of the sources using two strategies. First, we will examine cues that other research suggests convey objective quality of online information [44,46–48]: review credibility of additional sources linked to by the source, review credentials and affiliations of authors, identify any disclosure statements (financial disclosures, conflicts of interest), read any comments made by others to determine whether there is uncertainty or disagreement about the accuracy or appropriateness of the content, read any community notes and sources to which they may link (X only), examine the views-to-likes ratio (YouTube only), examine the likes-to-dislikes ratio (YouTube only). Second, we will modify the DISCERN instrument [49], which was designed to assess the quality of health information for consumers, to rate the quality of the sources.

The three team members conducting the online search will also perform the screening for credibility. Each will independently review credibility cues found for each source, score it using the DISCERN instrument, and then the three will discuss and form a final decision about the acceptability of a source for inclusion in the review. We will calculate weighted Fleiss’s kappa and use a cutoff score of 0.70 as an indicator of acceptable reliability among the three team members [50]. Details about the cues used to establish credibility and the DISCERN score will be added to the Excel file.

### Stage 4: Data extraction

Two team members will use Excel to complete the data extraction for the peer-reviewed articles and the online search. Data extraction will be an iterative process, with final categories decided upon as team members become more familiar with data and sources reviewed [35]. Data extracted will include, but is not limited to, author(s) name(s), year, demographics (e.g., age, sex, race) of autistic adolescents, setting, country, and modality. We will also extract whether details about accommodations to increase participation and engagement in FGs for autistic adolescents are described and, if so, the steps taken and any insight into the feasibility and acceptability of the accommodations that the source describes.

Each team member will independently extract data from a random sample of five included sources and compare results. Discrepancies will be discussed and, if needed, the categories used for data extraction will be revised. Conflicts will be resolved by a third team member. The process will be repeated until at least 75% agreement is reached between the two team members, at which point the remaining sources will be split between the two for data extraction.

For sources from the online search that included details about steps taken to make the FGs easier to participate for autistic adolescents, we will conduct an additional screening for credibility at this stage. We will conduct a search of peer-reviewed sources to determine whether research has evaluated the effectiveness of the steps described in the source to design other types of group settings (e.g., in classrooms, for therapy) that are empowering for autistic adolescents. Any evidence found that evaluates the effectiveness of a step will be added to the Excel file and described in the scoping review so that readers may make a determination.

Regarding ethics, Michigan State University’s Institutional Review Board does not consider the extraction of data from publicly available online sources as human subjects research. Nonetheless, we will take steps to enhance the ethical standards of our procedures. To protect the privacy of any minors whose content was collected in the online search, we will take steps to maintain the confidentiality of all content. We will de-identify the content by removing any personally identifying information such as profile pictures and usernames. As for the content itself, when reporting, we will use a method called “ungoogling” to maintain confidentiality [51]. Un-Googling means we will be paraphrasing content in a way that cannot be reverse-searched or linked back to the original user. We will never deposit the online content in a public site. Should a future researcher want to examine this material, they may contact Michigan State University’s Internal Review Board for access.

### Stage 5: Data analysis

To answer RQ1, we will create a bar chart to summarize the number of sources about autistic adolescents that included autistic adolescents in the FGs relative to other stakeholder groups. To answer RQ2–4, we will analyze only sources that included autistic adolescents in FGs. For RQ2, we will create a table of reported demographic characteristics and calculate descriptives of the sampled autistic adolescents who represent each characteristic. For RQ3, we will first create a bar chart to compare the number of sources that included versus omitted details regarding accommodations to make FGs accessible for autistic adolescents and then we will perform thematic analysis on the reported accommodations [52]. Example themes may include the target of the steps, such as the physical environment. To develop themes, two team members will independently develop codes and then discuss how to group them into themes. Each source may appear in multiple themes. We will create a table summarizing themes with examples of each theme from the sources. To answer RQ4, we will add to the table any details reported regarding the feasibility and acceptability of the accommodations. If no details are reported, we will also make note of this in the table.

### Stage 6: Consultation

We will consult with the neurodiverse team members and therapists who convene support groups for autistic adolescents about the results of our review. If they raise concerns about the appropriateness of a step suggested by a source, we will ask them for feedback on how to perform a search for evidence that raises skepticism about the step. We will incorporate this feedback into an additional search for evidence about the step. If we find evidence of raising skepticism about the step, we will exclude it from the review. If we do not, we will include it in our review but will note that concerns were raised during consultation.

### Limitations

Only sources written or translated to English will be included, thus excluding sources only available in other languages. This is a limitation reflects the language proficiencies available within our team. Because autism research is a global topic, limiting searches to English only may lead to some cultural bias, as various cultures may hold different views on autism and accommodations. The inclusion of online sources risks introducing bias, but we have developed a protocol to mitigate some risk.

## Discussion

Our planned review will identify the extent to which FGs about autistic adolescents are conducted with autistic adolescents. We will document inclusivity by recording participation, demographics, accommodations, and the feasibility and acceptability of the accommodations. FGs are an ideal method, particularly for autistic adolescents, for gathering input directly from those who stand to benefit from a project because incorporating their input early on enhances the likelihood that the project succeeds. Unfortunately, an initial literature review indicated that FGs *on* autistic adolescents are rarely conducted *with* autistic adolescents. Accordingly, we developed a protocol for a scoping review of methods used to make FGs inclusive for autistic adolescents that incorporates sources from non-academic outlets. Our goal is to leverage the size of our team, which includes undergraduate students and neurodiverse individuals, to mitigate risks of bias that may be introduced into the review from incorporating sources that have not been vetted by peer-review.

The review will summarize practices for designing FGs for autistic adolescents to increase their participation rates and engagement. The results of the review will be useful for researchers and practitioners from a range of fields, including design, education, health care, occupational health therapy, psychology, and public health. Other disability groups may benefit from inclusive design of FGs as well, because defaulting to these practices underpins anti-ableism [12,53]. Moreover, the methods we undertake in our scoping review may be useful for future scholarship seeking to incorporate results from Google Search and social media into their review and to partner with undergraduate students in the process.

## Supporting information

S1 Checklist PRISMA-P (Preferred Reporting Items for Systematic review and Meta-Analysis Protocols) 2015 checklist: recommended items to address in a systematic review protocol*. (PDF)
